# Reconstruction of the thumb interphalangeal joint from the second toe proximal interphalangeal joint combined with artificial dermis covering: A case report

**DOI:** 10.1097/MD.0000000000029043

**Published:** 2022-05-27

**Authors:** Haihua Wen, Weicong Deng, Yongchang Hong, Zhengjiang Lin

**Affiliations:** a Dongguan Hospital, Guangzhou University of Chinese Medicine, China; b Affiliated Dongguan Hospital, Southern Medical University, Dongguan, Guangdong Province, China.

**Keywords:** atificial dermi, case report, reconstruction, thumb interphalangeal joint, toe proximal interphalangeal joint

## Abstract

**Rationale:**

Thumb function is one of the most fundamental components of hand function, and a vast majority of hand functions are derived from thumb motion. Injury of the thumb interphalangeal joint has a tremendous impact on the function of the thumb, and damage to the thumb interphalangeal joint (IPJ) caused by trauma is usually accompanied by dislocation of the surrounding skin; therefore, it is particularly important to restore the thumb anatomy and skin coverage.

**Patient concerns:**

A 41-year-old woman presented with IPJ disfigurement accompanied by a local skin defect caused by machine compression of her right thumb. Restoring the appearance and function of the thumb is key to this operation.

**Diagnoses:**

Open fracture of the right thumb.

**Interventions:**

After detailed preoperative and radiographic evaluation, the appearance and function of the thumb were reconstructed by IPJ grafting and artificial dermis covering.

**Outcomes:**

At 4 months’ follow-up, the patient's visual analogue score was 0, no complications (eg, osteomyelitis, osteolysis, osteoarthritis, and nonunion of the artificial dermis) were observed, and the range of motion of the thumb IPJ returned to 60% of that of the healthy side.

**Lessons:**

The innovative application of the second toe proximal IPJ flap combined with double-layer artificial dermis covering to reconstruct the thumb IPJ defect not only solves the problem of skin defects in the recipient area after transplantation in previous cases but also restores the beauty of the recipient area, making it easier for patients to accept this surgical program.

## 1 Introduction

As the most commonly used part of people's daily lives, the hand is also the most vulnerable to injury, and its damage has the greatest impact on people's daily lives, especially the thumb. As the thumb accounts for 35% to 45% of the total function of the hand, Foucher ^[1–3]^ argues that damage to important finger joints, such as the proximal interphalangeal or metacarpophalangeal joints, is still more appropriate for preserving finger joint movement. Therefore, in cases of hand injury, thumb reconstruction is key to achieving functional recovery, including restoration of mobility, sensation, and esthetics.^[4]^ As a new material for covering complex skin and soft tissue defects, artificial dermi has the advantages of rapid growth and satisfactory postoperative appearance. At present, an increasing number of patients are willing to accept this material to treat complex wounds. On September 2, 2021 we admitted a case of thumb interphalangeal joint (IPJ) reconstruction using the proximal IPJ of the 2nd toe combined with a double layer of artificial dermal after a severe crush injury to the IPJ of the right thumb, with good results, which are reported below. The patient provided informed consent for publication of the case.

## 2 Case information

**Figure 1:**
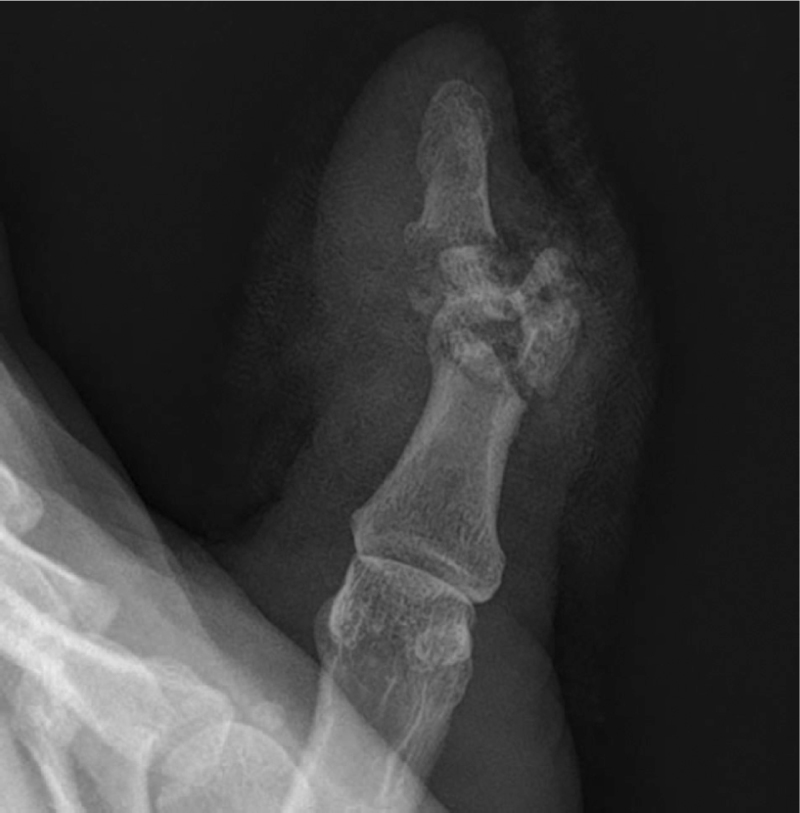
Preoperative oblique x-ray of the affected finger.

**Figure 2:**
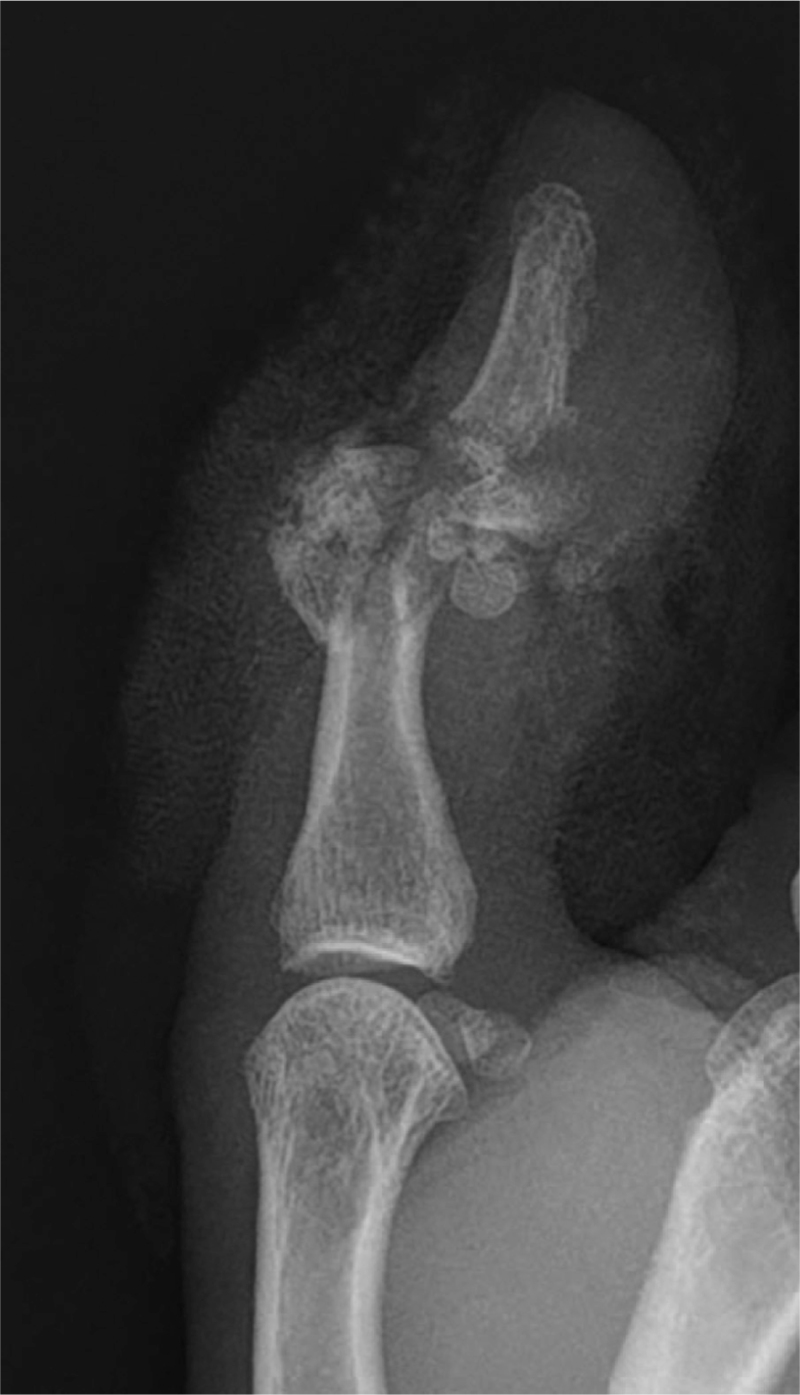
Preoperative x-ray of the affected finger.

The patient is female, 41 years’ old. She was admitted to the hospital with painful bleeding of the right thumb for 1 hour due to a machine crash injury. The patient was in good general condition, had no history of medical illness, and had no harmful habits of smoking or alcohol consumption. Physical examination revealed an irregular wound on the dorsal side of the IPJ of the right thumb with partial soft tissue loss, exposed bone, active bleeding, heavy contamination of the wound, inability to flex and extend, good extension and flexion of the metacarpophalangeal joint, and good wrist and palm function. Diagnosis: Open fracture of the right thumb. (Figs. 1 and 2)

**Figure 3:**
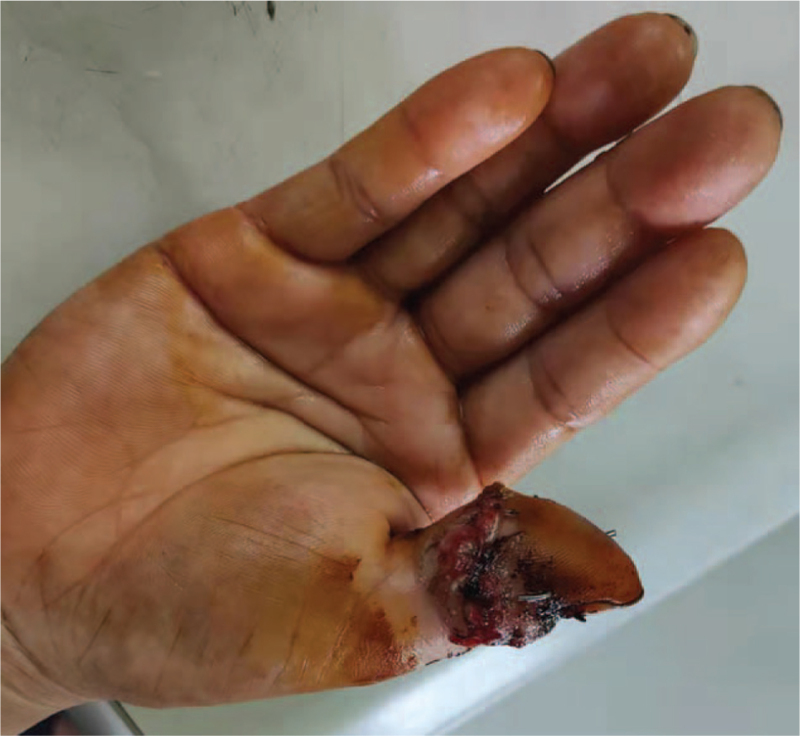
The appearance of the affected finger before the second surgery.

Due to severe contamination of the wound, the patient underwent emergency debridement of the right thumb and Kirschner wire fixation under local anaesthesia. After 4 days of treatment with dressing changes and intravenous cefazolin antibiotics (Fig. 3), the wound was observed to be devoid of infection and was sent to the operating theatre again for reconstruction of the IPJ of the right thumb with the 2nd toe proximal IPJ graft.

## 3 Surgical method

**Figure 4:**
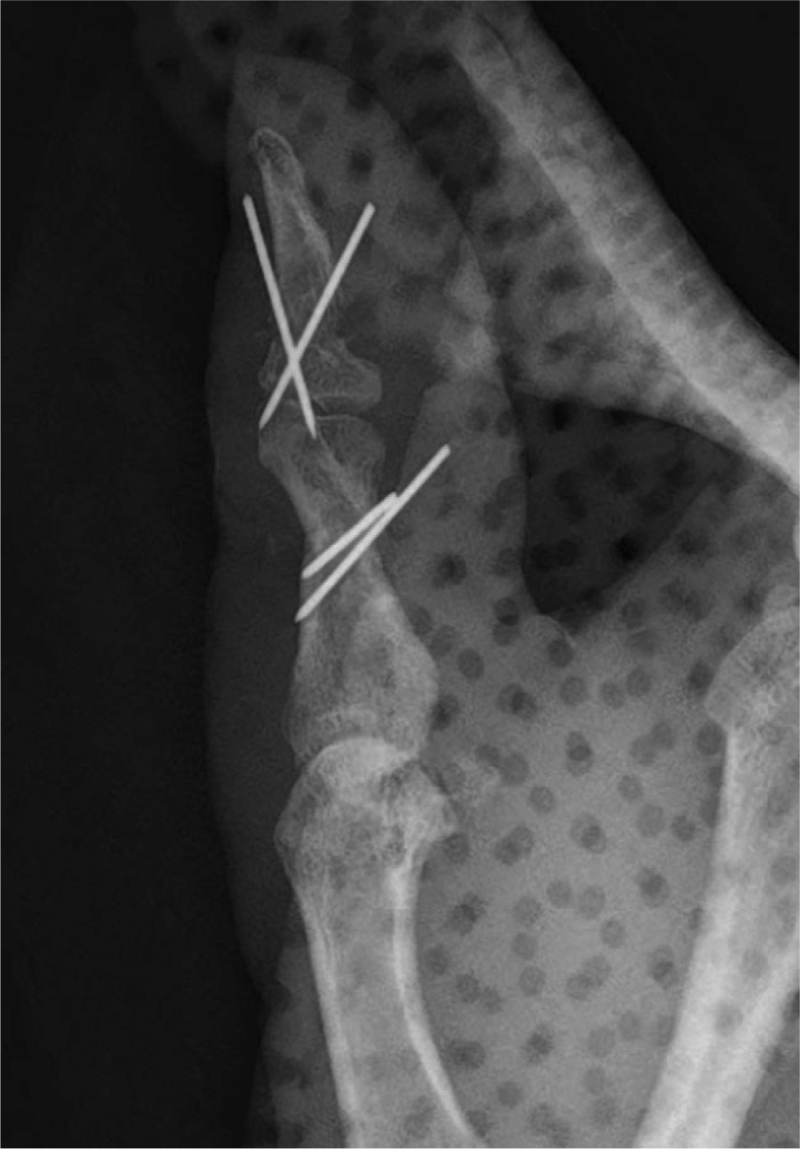
Postoperative x-ray of the affected finger.

**Figure 5:**
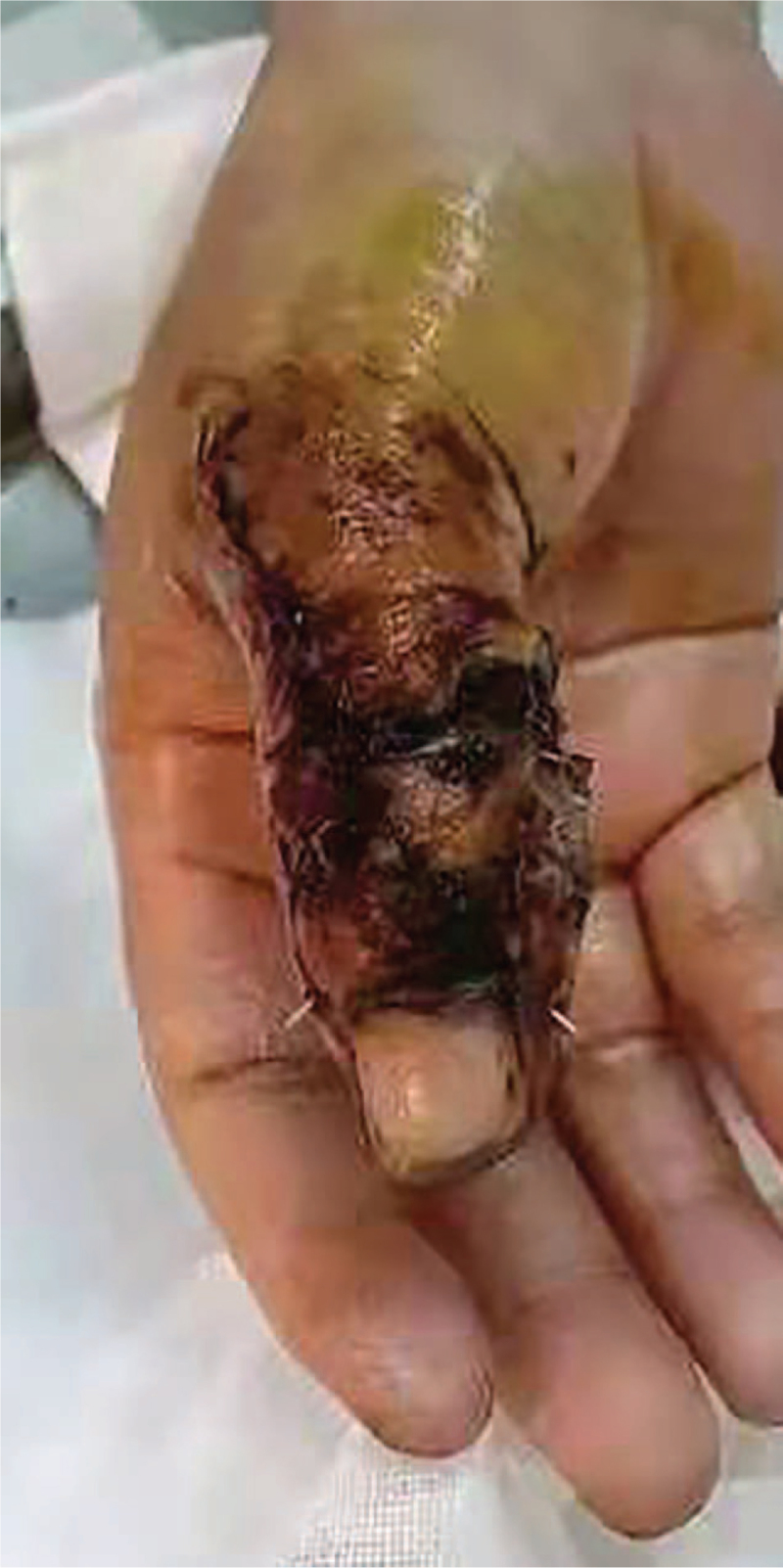
Postoperative appearance of the affected finger.

**Figure 6:**
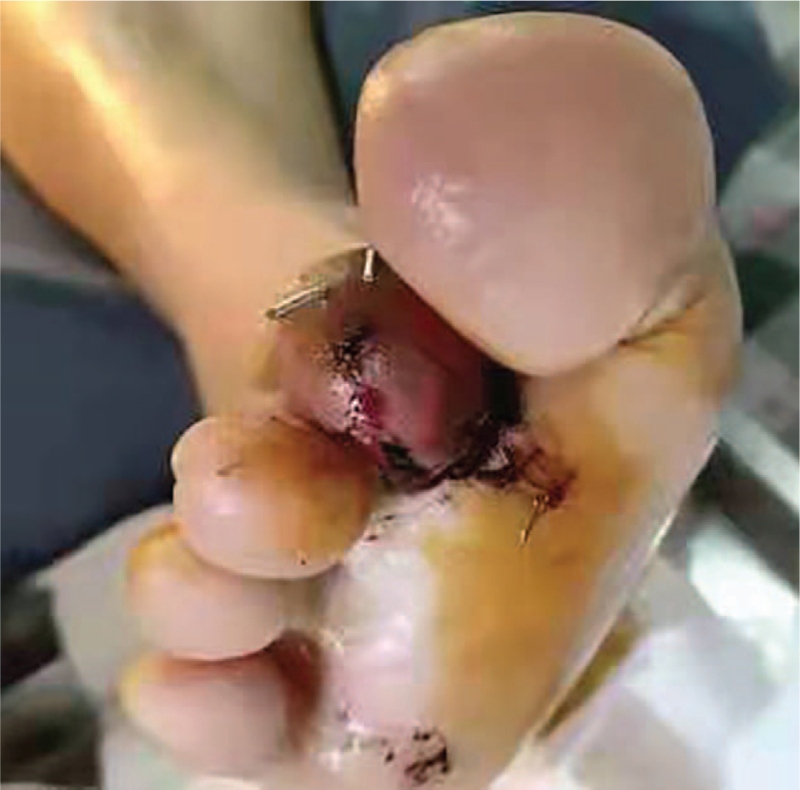
Postoperative appearance of donor toe.

**Figure 7:**
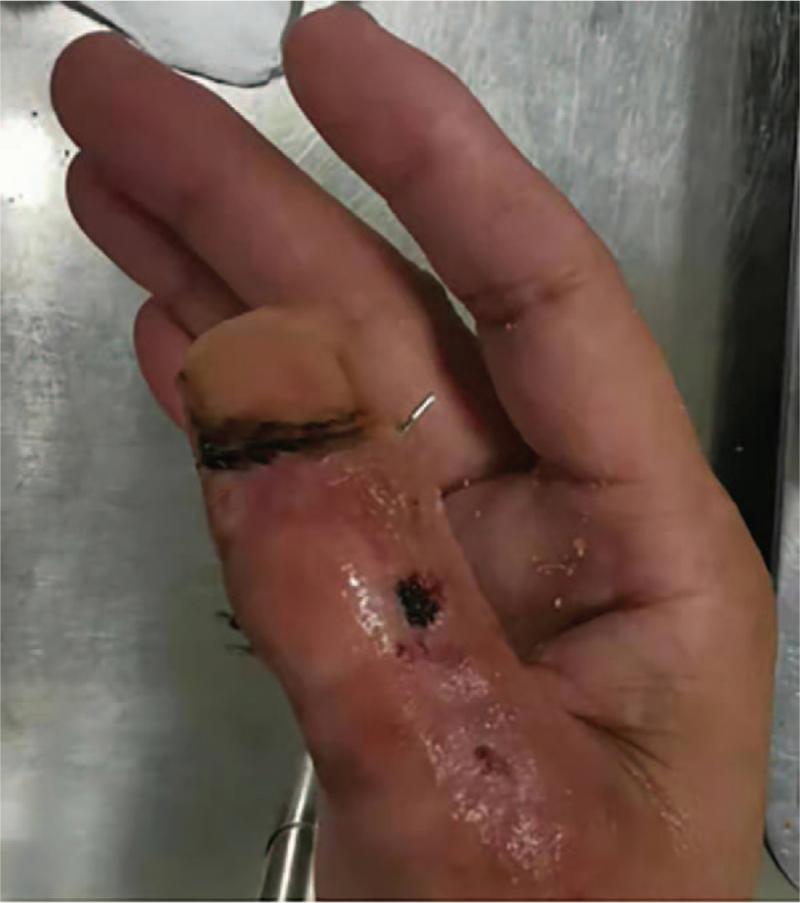
The appearance of the affected finger 2 months after surgery.

**Figure 8:**
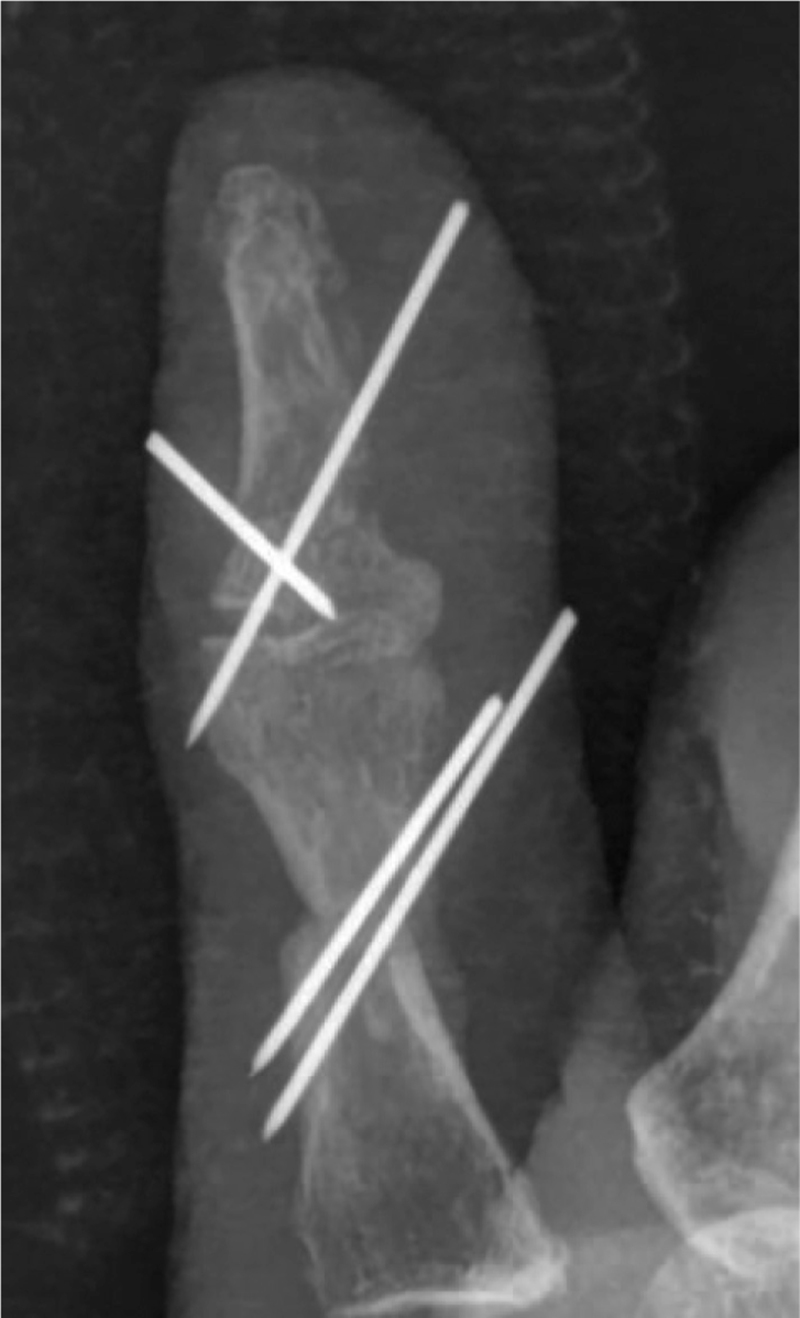
Orthopantomogram of the affected finger 2 months after surgery.

Surgery was performed under brachial plexus and lumbar anaesthesia. Surgery was performed under tourniquet control. Surgical design: According to preoperative radiographic measurements, the patient's thumb joint defect was 18 mm in length and 7 mm in width. On the basis of this measurement, a flap was designed on the tibial side of the proximal IPJ of the 2nd toe. Donor area preparation: The skin and subcutaneous tissue were cut according to the preoperative design line, preserving the intact joint capsule, periosteal tissue, and vascular reticular system; separate and cut the extensor and flexor tendons; carefully free the dorsal tibial artery of the 2nd toe; protect the branches pointing to the joint as far as possible; identify and cut the toe nerve; preserve the peroneal toe nerve and blood vessels and the plantar artery; cut the toe bone at both ends approximately 3 cm from the surface of the proximal IPJ, minimizing excision of the toe skin without affecting the blood flow of the joint flap, and freeing the 2^nd^ IPJ flap. The toe flexor and extensor tendons were repaired, the middle and proximal phalanges were crossed and fixed with Kirschner wires, the stump vessels were ligated, and the skin was trimmed and sutured. Preparation of the recipient area: Removing the Kirschner wire, making an incision along the original incision, removing the surface granulation, removing the surrounding free bone fragments, and amputating part of the distal and proximal phalanges of the thumb with an osteotome, leaving space for the toe proximal IPJ to be inserted. The radial phalangeal artery and dorsal phalangeal veins of the thumb were isolated and marked and the flexor tendon stop was preserved. Grafting: The free 2^nd^ proximal IPJ flap is trimmed, inserted into the interphalangeal space, and repositioned. The toe and phalanx were fixed with four 0.8 mm Kirschner wires, and the long extensor tendon and extensor tendon were trimmed and closed with a modified Kessler suture. The dorsal toe vein, dorsal thumb vein, dorsal toe artery, and finger artery were then anastomosed end-to-end under the microscope. After loosening the tourniquet, good blood flow was observed. After rinsing the wound, a skin defect of approximately 8 × 6 mm was observed on the dorsal side of the thumb, which was covered with artificial dermal material trimmed to the appropriate size. Finally, the wound was intermittently sutured and dressed (Fig. 4). Postoperative treatment included anti-infection, anticoagulation, anti-vascular spasm, swelling and pain relief, dressing changes, and external fixation of the hand in a plaster cast for two weeks, as well as appropriate postoperative elevation of the affected limb, wearing elastic stockings for two days, and prohibiting the foot from going to the ground for two weeks. One week postoperatively, the thumb metacarpophalangeal joint and wrist joint were given appropriate passive movement, and 15 days postoperatively, the silicone layer on the surface of the artificial dermis was removed, and passive movement of the thumb was enhanced (Figs. 5 and 6). Further rehabilitation was undertaken in a rehabilitation hospital after the removal of the kirschner wires 2 months after the operation (Figs. 7 and 8). Three months after surgery, x-rays showed bony healing. Four months after the second surgery, the VAS score was 0. No complications were observed (eg, osteomyelitis, osteolysis, osteoarthritis, and nonunion of artificial dermis), but there was still mild sensory disturbance in the thumb, and the mobility of the metacarpophalangeal and IPJs of the thumb was 0 to 60° and 0 to40°, respectively, which were 100% and 60% of the mobility of the contralateral side. The function of the opposite finger of the affected hand was normal and resumed normal work with very satisfactory results; the patient was satisfied with the present function and aesthetics of the affected finger.

## 4 Discussion

### 4.1 Surgical options for the repair of thumb defects

The choice of procedure for thumb reconstruction varies according to the plane of the injury. For distal segmental defects, reconstruction is usually performed using a toenail flap and adjacent finger flap; for mid-segmental defects, the main direction of repair is to restore the length of the thumb, which can be repaired using metacarpal lengthening, phalangeal reconstruction, and toe graft reconstruction^[5]^; for proximal segmental defects, toe grafting, index finger thumatization, and skin tube implant finger reconstruction can be used. Because of the importance of thumb to hand function, timely repair and reconstruction of the thumb can minimise disability and improve the patient's long-term quality of life. In this case, considering that the proximal IPJ of the second toe and the IPJ of the thumb are the closest in size, shape, and anatomy and are most compatible with the biomechanics of the IPJ of the thumb, the second proximal IPJ was excised to reconstruct the IPJ of the thumb.

## 5 Advantages of joint flap combined with artificial dermis for reconstruction

Metal, silicone, and coke are presently used as implants for the reconstruction of the IPJ of the finger. The use of a composite flap of the toe joint combined with artificial dermis covering to reconstruct the finger is not a common procedure in clinical practice, but it has many advantages over other choices.

Although silicone is presently the best choice for IPJ reconstruction, it has a postoperative complication rate of up to 11%, with the main complications being implant fracture, finger instability, and deformity.^[6–8]^ Some articles point out that the incidence of fracture 6.5 years after implantation is as high as 30%.^[9,10]^ At the same time, as the most widely used organ in people's daily lives,finger instability is limited to a large extent, especially in the index and middle fingers, where silicone spacers do not provide sufficient stability to support pressure on the thumb. Forster et al^[11]^ showed in a meta-analysis that the use of artificial implants would lead to more postoperative swan-neck deformities, which would severely affect the functional activity of the thumb. This may be related to the hinge design of the implants. Therefore, the use of artificial implants for IPJ reconstruction is uncommon in clinical practice. However, the use of a toe composite flap for thumb IPJ reconstruction is not associated with rejection, osteolysis, or other postoperative ruptures of the graft, and the repair of the damaged tendon during the composite flap graft, together with the reinforcement of the joint stability by the collateral ligament on the healthy side, significantly reduces the incidence of postoperative finger instability and deformity complications.^[12]^

The artificial implant strips all blood vessels around the joint, destroying the skeletal nutrient vessels and blocking bone development. The composite flap acts as a joint with vascularised bone, preserving the potential for continued bone development in patients whose bones are still mature.^[13]^ After the composite flap has been grafted, the microscopic vessels within it can gradually crawl and repair, restoring the nutritional vessels needed for bone growth and providing a better surgical option for patients with immature bones. The composite flap carries abundant blood vessels and sufficient bone at the time of grafting for rapid bone healing, which can slow down joint degeneration and facilitate early functional exercise of the thumb.

The most important clinical concern for patients with partial loss of the second toe is whether it will have a significant impact on future ambulation. It has been documented that the ground reaction force of the second toe with a reconstructed bunion is not different from that of a normal foot.^[14]^ In terms of human dynamics, the plantar pressure on the first metatarsal head is the greatest during both walking and running. This demonstrates that the absence of the second toe does not have a serious impact on daily life, which is very convincing in addressing the patient's preoperative concerns regarding the change in walking after 2^nd^ toe loss. The thumb accounts for 35% to 45% of the total function of the hand, and the need to use the thumb in daily life is much greater than the need to use the second toe, which is a procedure that has more advantages than disadvantages for patients.

Skin coverage after joint reconstruction is particularly important. Conventional surgery carries the skin of the toe for reconstruction, which is suitable for wounds that are relatively well-defined and where the surrounding skin is in fair condition. The double-layered artificial dermis, with a semipermeable silicone membrane on top and a porous matrix of depsipeptide bovine Achilles tendon collagen and glycosaminoglycan at the bottom, has good long-entry properties, making it relatively simple to cover complex wounds, significantly shortening the operation time, and reducing skin scarring in the donor area.^[15]^ In addition, the double-layer artificial dermis eliminates the need for secondary skin grafts for smaller wounds, further reducing hospital stay. This results in better function and better postoperative appearance for the patient, which increases confidence in postoperative recovery.

## 6 Details to note during this operation

In this procedure, dissection and freeing of the dorsal arterioles and nerves are crucial to the success or failure of the toe graft. Therefore, careful separation of the dorsal arterioles and nerves on the required side is necessary during the preparation of the donor area, while care should be taken to protect the intrinsic plantar arterioles and nerves on the same side to ensure the survival of the donor toe. If necessary, microscopic separation should be used to reduce damage and preserve toe joint nutrition and sensation as much as possible, while ensuring adequate vascular and neurological nutrition of the joint graft. This may help delay toe degeneration. After separation, the vascular nerve of the thumb was sutured to the vascular nerve of the toe graft with a 10-0 or smaller suture under a microscope.

The IPJ of the toe is a trochlear joint, which is slightly flexed at rest, approximately 20 to 30 degree, whereas the IPJ of the thumb is in extension or slightly flexed at 5 degree at rest. Therefore, it is necessary to correct toe flexion when transplanting a joint. The assistant should keep the IPJ in the hyperextended position as far as possible during the operation, and by adjusting the tension of the tendon suture, the tension should be greater when the bunion extensor tendon is sutured to the toe extensor key, and less when the bunion flexor key and toe flexor key are sutured, so that the reconstructed IPJ changes from a flexed to an extended position at rest ^[16]^ to prevent the occurrence of insufficient extensor finger or mallet finger phenomenon during long-term follow-up.

Patients undergoing this procedure usually have poor skin conditions in their hands, with varying degrees of soft tissue and tendon defects. In previous surgical cases, large flaps were usually used to prevent incision closure difficulties and vascular compression, which led to large differences in the tibiofibular and fibular skin of the donor toe, resulting in swelling of the toe and affecting the appearance of the donor toe.^[17]^ In this case, due to the poor skin condition of the bunion, which is very easy to tear, a double layer of artificial dermis was used to cover the wound, which not only makes the wound more beautiful, but also does not affect the postoperative blood transport observation, and has clinical safety.

Not only does joint reconstruction require the same static activity as a normal thumb joint, but the restoration of motor function is also equally important, placing high demands on the motor and stabilisation systems of the graft joint, which requires the surgeon to have extensive experience in this operation. However, the clinical results are unsatisfactory. In a study with a mean follow-up of 42 months,^[18]^ the average flexion arc of the IPJ after reconstruction was 43 degree, which is less than half of the 90 degree arc of the normal IPJ. However, this is also related to the stiffness of the joint due to prolonged postoperative immobilisation. This requires the surgeon to fully communicate with the patient before surgery and appropriately reduce postoperative expectations. In terms of postoperative exercise, it has been documented that prolonged braking of the IPJ after reconstruction can result in progressive extensor function loss.^[19]^ There is a lack of academic guidance regarding when to perform any type of functional exercise. Reconstruction of the IPJ of the thumb is more delicate than reconstruction of other large joints, and requires a high level of resources and experience from the surgical team.

## Author contributions

**Conceptualization:** Haihua Wen, Weicong Deng, Yongchang Hong, Zhengjiang Lin.

**Data curation:** Haihua Wen, Weicong Deng, Yongchang Hong, Zhengjiang Lin.

**Formal analysis:** Weicong Deng, Yongchang Hong, Zhengjiang Lin.

**Methodology:** Haihua Wen, Weicong Deng, Yongchang Hong.

**Project administration:** Haihua Wen, Weicong Deng, Yongchang Hong.

**Writing – original draft:** Weicong Deng.

**Writing – review & editing:** Haihua Wen, Yongchang Hong, Zhengjiang Lin.
